# Insect-Resistant Variety *Populus deltoides* ‘Shalinyang’ May Decrease *Anoplophora glabripennis* Females’ Fecundity by Suppressing the Serine/Threonine Kinase *AglaAkt* Gene

**DOI:** 10.3390/insects17030250

**Published:** 2026-02-27

**Authors:** Hui-Quan Sun, Yu-Jun Kong, Qiu-Mei Zhong, Xin-Yi Liu, Fei-Fei Cui, Jian-Feng Liu, Zhi Su, Jian-Rong Wei

**Affiliations:** 1 School of Life Sciences/Hebei Basic Science Center for Biotic Interaction, Hebei University, Baoding 071002, China; hhsunhq@163.com (H.-Q.S.); kongkong0701@163.com (Y.-J.K.); zhong_qm0728@163.com (Q.-M.Z.); m18260813258@163.com (X.-Y.L.); cuifeifei7810129@126.com (F.-F.C.); jianfengliu@hbu.edu.cn (J.-F.L.); 2 Engineering Research Center of Ecological Safety and Conservation in Beijing-Tianjin-Hebei (Xiong’an New Area) of MOE, Baoding 071002, China; 3 Experimental Center of Desert Forestry, Chinese Academy of Forestry, Dengkou 015200, China; slzxsuzhi@163.com

**Keywords:** *Anoplophora glabripennis*, transcriptomic, serine/threonine kinase, reproduction, insect-resistant poplar, plant-insect interaction, RNA interference

## Abstract

*Anoplophora glabripennis* Motschulsky (Coleoptera: Cerambycidae) is notorious for its wide host range, serious damage caused, and the difficulties involved in controlling it. *Populus deltoides* ‘Shalinyang’ (PdS) has demonstrated a strong inhibitory effect on the larval growth and the adult lifespan of *A. glabripennis* and can be used as an “attract-and-kill” tree in mixed shelterbelt construction. However, how *A. glabripennis* react to this resistant poplar remains unclear. This research employed transcriptomics techniques to study transcriptional responses in the midgut of *A. glabripennis* after consuming PdS, *Elaeagnus angustifolia* L. (EA), and *Salix matsudana* Koidz (SM), respectively. One of the key findings revealed that the PIK3-Akt signaling pathway was suppressed in *A. glabripennis* feeding on PdS compared with those feeding on EA or SM. Further RNA interference results demonstrated that silencing the *AglaAkt* gene significantly decreased the levels of AKT, phospho-AKT, vitellogenin, vitellogenin receptor, and vitellin in *A. glabripennis*. Therefore, we speculate that the *AglaAkt* gene may be an underlying target gene that causes the low reproductive capacity of *A. glabripennis* when feeding on PdS. This finding provides important insights into the role of the *AglaAkt* gene in mediating the reproductive development in *A. glabripennis* and its molecular response to the resistant poplar.

## Simple Summary

The Asian longhorned beetle (ALB), *Anoplophora glabripennis* Motschulsky (Coleoptera: Cerambycidae), is a highly destructive wood-boring pest that causes significant damage to poplar trees in farmland shelterbelts across Northwest China. *Populus deltoides* ‘Shalinyang’ (PdS) is a new stress-resistant poplar variety developed by the Chinese Academy of Forestry and shows resistance to ALB. It can be used as an “attract-and-kill” tree in mixed shelterbelt construction. However, the mechanisms behind PdS’s effects on the growth, development, and reproduction of ALB are not fully understood. In this study, transcriptomic analysis and quantitative real-time PCR showed that the serine/threonine kinase *AglaAkt* gene expression in the midgut of ALB females fed on PdS was significantly lower than in those fed on either *Elaeagnus angustifolia* L. or *Salix matsudana* Koidz. Subsequently, RNA interference-mediated silencing of *AglaAkt* resulted in decreased vitellogenin production and impaired vitellin deposition in female beetles. These results suggest that *AglaAkt* may be a key target gene involved in the response to PdS and may contribute to the reduced fecundity observed in ALB.

## 1. Introduction

Herbivorous insects and their host plants are closely linked, with insects relying on these plants throughout parts of their life cycle for growth, development, emergence, and reproduction [1,2]. The quality of the host plant also affects herbivorous insects’ immune defenses, diapause, fecundity, and the number of generations they produce each year [3,4,5]. Among these aspects, the reproductive process of female insects has attracted particular interest because of its crucial role in species survival and its potential as a target for pest control [6,7,8,9].

The reproductive process of female insects requires large amounts of nutrients and energy. Given the close relationship between host plants and herbivorous insects, host plants likely represent one of the strongest selective pressures driving the evolution of reproductive traits in these insects [1]. In other words, host plant quality—such as the availability of carbon, nitrogen, and defensive compounds—is a major factor affecting herbivorous insect fecundity [4]. Recent studies show that the Target of Rapamycin (TOR) and insulin signaling pathways act as nutritional checkpoints that control insect reproduction [10,11,12]. Akt, a key regulator in the intracellular insulin signaling cascade, not only transmits insulin signals by phosphorylating downstream targets but also activates the TOR pathway by inhibiting the TSC1/TSC2 complex and PRAS40 [10,13,14,15]. Furthermore, an increasing number of studies have emphasized the crucial role of Akt in regulating reproductive development across various pest species [16,17,18,19,20]. These studies used RNA interference (RNAi) to reduce *Akt* gene expression in pests like *Tribolium castaneum* (Herbst) (Coleoptera: Tenebrionidae), *Lasioderma serricorne* (F.) (Coleoptera: Anobiidae), and *Frankliniella occidentalis* (Pergande) (Thysanoptera: Thripidae), showing that lower expression of the *Akt* gene decreases fecundity by blocking ovarian development and vitellogenin gene expression [6,21,22,23]. These findings suggest that targeting *Akt* or related genes could be an effective, green, and sustainable strategy for controlling insect populations.

The Asian longhorned beetle (ALB), *Anoplophora glabripennis* Motschulsky (Coleoptera: Cerambycidae), is notorious for its wide host range, serious damage, and difficulty of being controlled [24,25]. In China, it is a major threat to poplar trees in the northwest part of the “Three-North Shelterbelt” [26,27,28,29,30]. Currently, researchers have carried out comprehensive studies on its management strategies, such as screening for natural enemies, breeding resistant tree varieties, and applying different silvicultural control methods [29,31,32,33,34]. Among these, breeding, screening, and applying resistant varieties are prerequisites for establishing insect-resistant mixed forests with self-regulating capabilities. An increasing number of varieties have been identified as resistant to ALB, including *Elaeagnus angustifolia* L. (EA), *Populus tomentosa* Carrière, *P. alba* var. *pyramidalis* Bunge, *P. deltoides* cv. ‘Lux’ (I-69/55), *P. deltoides* cl. Nankang-1 and *P. deltoides* ‘Shalinyang’ (PdS) [28,30,31,35,36,37]. However, varieties such as *P. tomentosa*, *P. deltoides* cv. ‘Lux’ (I-69/55), and *P. deltoides* cl. Nankang-1 are difficult to promote and apply in Northwest China because of insufficient cold or drought tolerance [31]. It is worth noting that both PdS and EA can be considered “attract-and-kill” tree species for ALBs because they can both attract ALBs to lay eggs and cause the larvae to die [31,37,38]. Notably, PdS, a novel stress-resistant poplar cultivar for Northwest China bred by the Chinese Academy of Forestry, is identified as an ALB-resistant variety for ALB control due to its extremely high larval mortality [31,37,39,40,41]. In addition, we found that PdS has a strong inhibitory effect on the lifespan and fecundity (female egg production) of ALB adults [37,39]. Specifically, ALB adults that feed on PdS have shorter lifespans and lower fecundity than those that feed on EA or *Salix matsudana* Koidz (SM), with EA-fed ALB adults having the longest lifespan and the highest fecundity. Therefore, our previous findings suggest that EA is not a suitable choice for controlling this beetle during shelterbelt construction, as it may provide ample nutrition conditions for adult ALBs and increase their lifespan and fecundity [37]. However, the regulatory mechanisms by which PdS or EA influence the growth, development, and reproduction of female adults remain poorly understood. In the context of pest control, the mechanism of action of PdS on ALBs is a particularly worthwhile question to explore, especially compared with other plants.

To investigate the impact of PdS and EA—two “attract-and-kill” species from the distinct genera *Populus* and *Elaeagnus*, respectively—on female ALB reproduction, we performed transcriptome analysis of the midguts of female adults that fed on these three trees, using SM (a conventional host from the genus *Salix*) as the control. Subsequently, we analyzed differentially expressed genes (DEGs) in the midguts of female adults after feeding on different plants and used quantitative RT-PCR (qRT-PCR) to verify the expression patterns of key genes across plants. Furthermore, to exploit the favorable trait of PdS, which leads to low ALB fecundity, the role of *AglaAkt* in the reproductive regulation of ALB was investigated through RNA interference (RNAi) and Enzyme-linked Immunosorbent Assay (ELISA). Our findings provide a foundation for understanding the resistance mechanisms of PdS against ALBs, clarifying the differential effects of the two “attract-and-kill” tree species on ALBs and identifying potential target genes for controlling this pest.

## 2. Materials and Methods

### 2.1. Insect Preparation

ALB adults used in this study were purchased from the Institute of Forest Ecology, Environment and Nature Conservation, Chinese Academy of Forestry. Newly emerged adults were individually placed in a transparent plastic container (18 cm × 11 cm × 8 cm, PE) for 48 h and no food was provided.

The three tree species used, which produce different effects on ALBs, are common in the Hetao Plain region of Inner Mongolia. The PdS, EA, and SM trees used in this study were from the Experimental Center of Desert Forestry, Chinese Academy of Forestry and planted in School of Life Sciences, Hebei University. One-year-old potted seedlings that were uniformly watered and pesticide-free were inoculated with ALB adults. Each seedling was set in a sieve net cage and two ALB adults (one male and one female) were introduced for feeding. ALB adults feeding on EA and SM served as controls. Ten biological replicates were conducted for each tree species.

To investigate the effects of three tree species on the development and reproduction of ALB adults, according to our previous study, females feeding on a single host tree that reached 70% of their average lifespan were sampled. Among them, the female ALBs that fed on PdS were 12 days old, the female ALBs that fed on SM were 22 days old, and the female ALBs that fed on EA were the oldest at 42 days old. The tissues of female adults were dissected on a clean bench, frozen in liquid nitrogen, and stored at −80 °C for subsequent analysis.

### 2.2. RNA Extraction, cDNA Library Construction and Transcriptome Sequencing

Total RNA was extracted from the midgut of ALB females using the RNApure Fast Tissue & Cell Kit (CW0599S, CWBIO, Taizhou, China), with three biological replicates for each tree species. The concentration of total RNA was measured with a NanoDrop spectrophotometer (ThermoFisher Scientific, Waltham, MA, USA), and the purity was checked on a 1.2% agarose gel.

The RNA-seq transcriptome library was prepared using Illumina^®^ Stranded mRNA Prep, Ligation (San Diego, CA, USA) with 1 μg of total RNA by Shanghai Majorbio Bio-pharm Bio-technology Co., Ltd. (Shanghai, China). After quantification with Qubit 4.0, the library was sequenced on the NovaSeq™ X Plus platform (Illumina, San Diego, CA, USA), producing nine high-quality sequencing libraries.

### 2.3. Transcriptome Assembly and Data Analysis

The obtained raw data were filtered using fastq (version 0.23.4) with default parameters [42]. After removing low-quality raw reads, the clean reads were mapped to the ALB reference genome (https://www.ncbi.nlm.nih.gov/datasets/genome/GCF_000390285.2/, accessed on 5 November 2024) using HiSat2 (version 2.2.1) [43]. Transcript assembly was conducted with Cufflinks (version 2.2.1) [44], and gene expression levels were measured in Transcripts Per Million (TPM) using RSEM (version 1.3.3) [45]. Differential expression analysis was then carried out with DESeq2 (version 1.42.0) [46]. The screening criteria for differentially expressed genes (DEGs) were set to meet |log2FC| ≥ 1 and false discovery rate (FDR) < 0.05. To better understand the biological functions of these DEGs, Goatools (version 1.4.4) [47] and Python scipy packages (http://scipy.org/install/, accessed on 9 November 2024) were used to perform Gene Ontology (GO, http://geneontology.org/) terms and Kyoto Encyclopedia of Genes and Genomes (KEGG, http://www.kegg.jp/) pathway enrichment analyses on DEGs, respectively.

### 2.4. Gene Expression Quantification Assay

The relative expression levels of candidate genes in the midguts of ALB female fed EM or SA for identical feeding times (12 days old, grouped as SMMG-IFM and EAMG-IFM) as those fed PdS were analyzed using the Roche LC96 Real-Time PCR System (Roche Diagnostics, Indianapolis, IN, USA), and the relative expression levels of candidate genes in the transcriptome were also verified. cDNA templates were synthesized from total RNA after genomic DNA removal using the HiFiScript All-in-One RT Master Mix for qPCR Kit (CW3371, CWBIO, Taizhou, China) according to the manufacturer’s instructions. Each qRT-PCR reaction (10 μL) contained 5 μL of 2 × TB Green Premix Ex Taq (Tli RNaseH Plus) (Takara, Dalian, China), 0.4 μL of each primer (10 μM), 0.8 μL of cDNA template (500 ng), and nuclease-free water to adjust the final volume. Thermal cycling conditions were as follows: initial denaturation at 95 °C for 2 min, followed by 45 cycles for 5 s at 95 °C, 58 °C for 30 s, and 72 °C for 30 s. Raw RT-PCR data were acquired using Lightcycler 96 SW 1.1. Subsequently, the transcript levels of target genes were normalized to the geometric mean of reference genes and calculated using the 2^−ΔΔCT^ method [48]. All reactions were performed with three biological replicates, and three technical replicates were performed for each replicate. All specific primers are listed in Table S1.

### 2.5. Double-Strand RNA (dsRNA) Synthesis and Injection

To obtain specific dsRNA for the serine/threonine kinase *AglaAkt* gene of ALB, in vitro transcription was performed using the T7 RNAi transcription kit (Cat. No. TR102-02, Vazyme, Nanjing, China) according to the manufacturer’s instructions. A dsRNA control (ds-*EGFP*) was provided by Shanghai Plant Science Biotechnology Co., Ltd. (Shanghai, China). The dsRNA-specific primers used in this study were designed in dsRNAEngineer [49] (https://dsrna-engineer.cn/) and listed in Table S1.

Ten-day-old female ALBs fed with EA to ensure sexual maturity were selected as test insects. Approximately 5 μg of *ds-AglaAkt* was injected into each female ALB using a microinjector (Shanghai Gaoge Industry and Trade Co., Ltd., Shanghai, China). Female ALBs injected with enhanced green fluorescent protein (ds-*EGFP*) served as the negative control. After dsRNA injection, both the control and experimental groups of ALB continued to be fed EA. Three individuals were collected 24, 48, 72, and 120 h after dsRNA injection. RNAi efficiency was assessed by qRT-PCR, and three biological replicates were prepared. At 120 h after dsRNA injection, the midgut, ovaries, and hemolymph of adult females were collected. The levels of AKT protein/kinase (AKT), phospho-AKT (p-AKT), vitellogenin (VTG), vitellogenin receptor (VgR), and vitellin (Vn) in ALBs were measured using ELISA kits (Meilian Biotechnology Co., Ltd., Shanghai, China).

### 2.6. Statistical Analysis

All data were analyzed using Graphpad Prism 10.5 and presented as mean ± standard error (SE). Differences between treatments were compared using Student’s *t*-test, with significance levels of * *p* < 0.05, ** *p* < 0.01, and *** *p* < 0.001. Differences between multiple groups were compared using one-way analysis of variance (ANOVA) with multiple comparisons performed using Tukey’s test.

## 3. Results

### 3.1. RNA Sequencing and DEG Analysis

**Figure 1:**
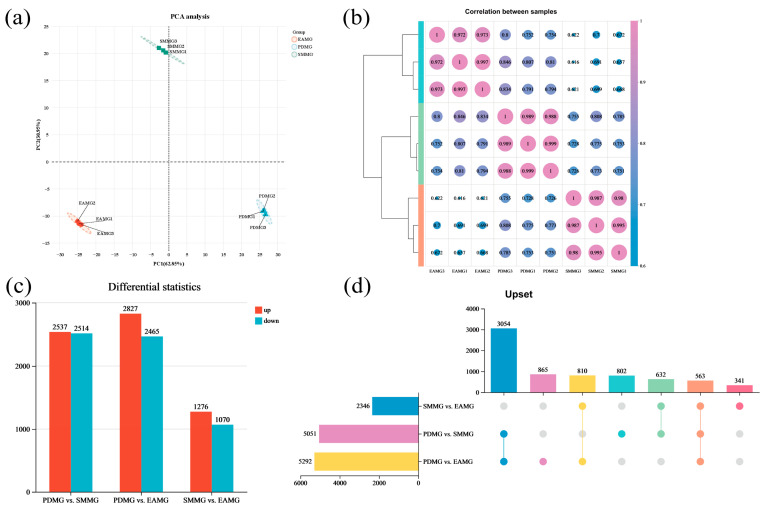
Overall analysis of transcriptome data. (**a**) Principal component analysis (PCA) between different samples; (**b**) correlation heatmap between different samples; (**c**) overview of the differentially expressed genes (DEGs) among different treatment groups; (**d**) upset analysis of the DEGs among different treatment groups.

A total of 416,122,034 raw reads were initially obtained. After filtering and error checking, 413,298,224 high-quality reads from nine samples were used for subsequent bioinformatics analysis. All samples met high-quality standards, with an average GC content exceeding 44%, and Q20 and Q30 percentages exceeding 98.65% and 95.92%, respectively. Furthermore, over 84.82% of the sequences were successfully aligned to the ALB genome, and uniquely aligned sequences accounted for 71.14–85.51% of the total aligned sequences, confirming the reliability of the sequencing data (Table 1 and Figure S1). PCA based on TPM values showed that all biological replicates were well grouped and separated (Figure 1a), and Pearson correlation analysis showed that there was a high degree of correlation between the samples (Figure 1b).

To assess the relative gene expression levels in the midgut of female ALBs feeding on different trees, we used DESeq2 (version 1.42.0) to analyze and identify DEGs among three comparison groups (PDMG vs. SMMG, PDMG vs. EAMG, and SMMG vs. EAMG). A total of 7067 DEGs were identified (Table S2), and they are displayed according to how they are distributed across the comparison groups: PDMG vs. SMMG (5051 DEGs: 2537 upregulated and 2514 downregulated), PDMG vs. EAMG (5292 DEGs: 2827 upregulated and 2465 downregulated), and SMMG vs. EAMG (2346 DEGs: 1276 upregulated and 1070 downregulated) (Figure 1c). There were 563 common DEGs across all three comparison groups (Figure 1d).

### 3.2. GO and KEGG Pathway Analysis of DEGs

To understand the biological functions of DEGs, we first analyzed all DEGs in the three comparison groups using the GO annotation system. The results showed that all DEGs of the three comparison groups were divided into 38 level-2 functional classification terms including 18 biological processes, 2 cellular components, and 18 molecular functions (Figure S2 and Table S3). The top three GO terms for the DEGs were metabolic process (GO:0008152), cellular process (GO:0009987), and biological regulation (GO:0065007) for biological process; and catalytic activity (GO:0003824), binding (GO:0005488), and transporter activity (GO:0005215) for molecular function, respectively. In contrast, in the cellular component, the DEGs were exclusively annotated to just two GO terms: cellular anatomical entity (GO:0110165) and protein-containing complex (GO:0032991).

**Figure 2:**
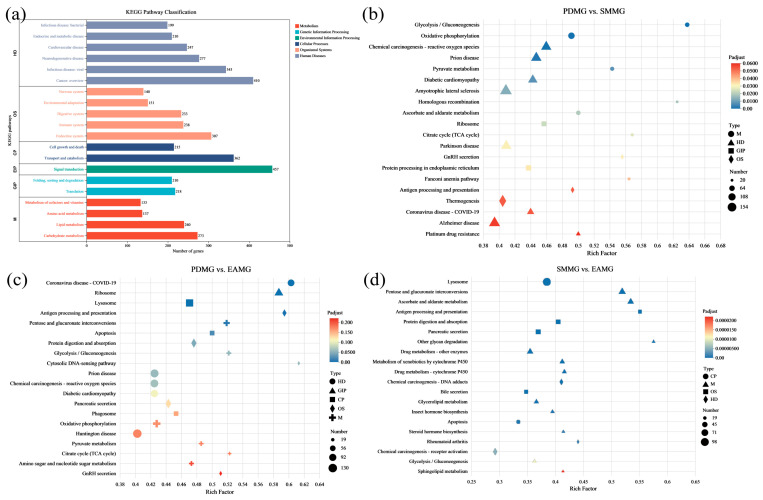
Kyoto Encyclopedia of Genes and Genomes (KEGG) pathway analysis of the DEGs among different treatment groups. (**a**) Statistical analysis of KEGG classification of all DEGs across different treatment groups; (**b**) statistical analysis of the enrichment of the top 20 KEGG pathways of the DEGs between PDMG vs. SMMG. The *x*-axis represents the level of Rich factor, and the *y*-axis represents the type of pathway. Dot size represents the number of DEGs, and colors indicate corrected *p*-values. The shape of the dot represents the KEGG classification, specifically as follows: M: Metabolism, GIP: Genetic Information Processing, CP: Cellular Processes, OS: Organismal Systems, HD: Human Diseases. The same applies to graphs c and d; (**c**) statistical analysis of the enrichment of the top 20 KEGG pathways of the DEGs between PDMG vs. EAMG; (**d**) statistical analysis of the enrichment of the top 20 KEGG pathways of the DEGs between SMMG vs. EAMG.

Moreover, DEGs were classified into six subsections by KEGG pathway, including metabolism, genetic information processing, environmental information processing, cellular processes, organismal systems, and human diseases (Figure 2a). Subsequently, KEGG enrichment analysis was performed on the DEGs in the three comparison groups. The results showed that DEGs in PDMG vs. SMMG, PDMG vs. EAMG, and SMMG vs. EAMG were significantly enriched with 18, 7, and 61 KEGG pathways, respectively (Table S4). Among them, only the SMMG vs. EAMG group showed significant enrichment of the environmental information processing category of the KEGG pathway. To reveal the mechanism by which PDS affects the growth and development of female ALBs, we analyzed DEGs in the KEGG pathway that were significantly enriched in two comparison groups: PDMG vs. SMMG and PDMG vs. EAMG (Figure 2b–d). Interestingly, the *PIK3* and *Akt* genes (PIK3-Akt signaling pathway) were annotated in two pathways: “apoptosis” (PDMG vs. EAMG) and “chemical carcinogenesis-reactive oxygen species” (PDMG vs. SMMG). It is noteworthy that the expression levels of these two genes were significantly lower in the midgut of beetles fed with PdS than in those fed with the other two tree species.

### 3.3. Differential Expression Analysis of the AglaAkt Gene in Different Treatment Groups

**Figure 3:**
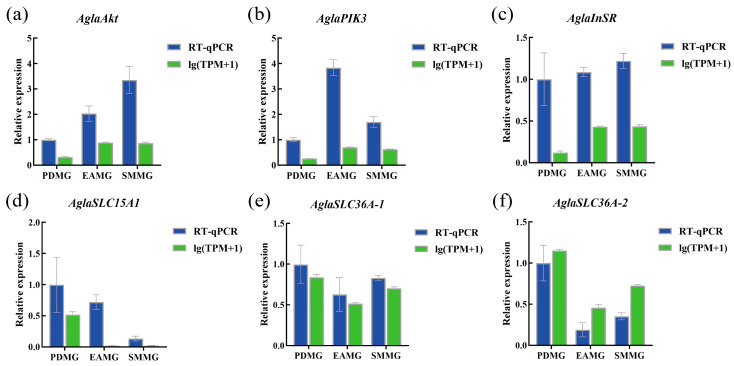
Quantitative RT-PCR (qRT-PCR) validation of six differentially expressed genes chosen from the transcriptome. (**a**) The serine/threonine kinase *AglaAkt* gene; (**b**) the phosphatidylinositol 4,5-bisphosphate 3-kinase *AglaPIK3* gene; (**c**) the insulin receptor *AglaInSR* gene; (**d**) the solute carrier *AglaSLC15A1* gene; (**e**) the solute carrier *AglaSLC36A-1* gene; (**f**) the solute carrier *AglaSLC36A-2* gene. Data are expressed as mean  ±  SE of three biological replicates.

In addition to *AglaPIK3* and *AglaAkt*, we selected four genes (*AglaInSR*, *AglaSLC15A1*, *AglaSLC36A-1*, and *AglaSLC36A-2*) at random to corroborate the authenticity of the RNA-seq findings. The relative expression levels of these genes, as determined by qRT-PCR, are shown in Figure 3. The qRT-PCR results were consistent with the RNA-seq results, indicating that the RNA-seq data were accurate and reliable.

**Figure 4:**
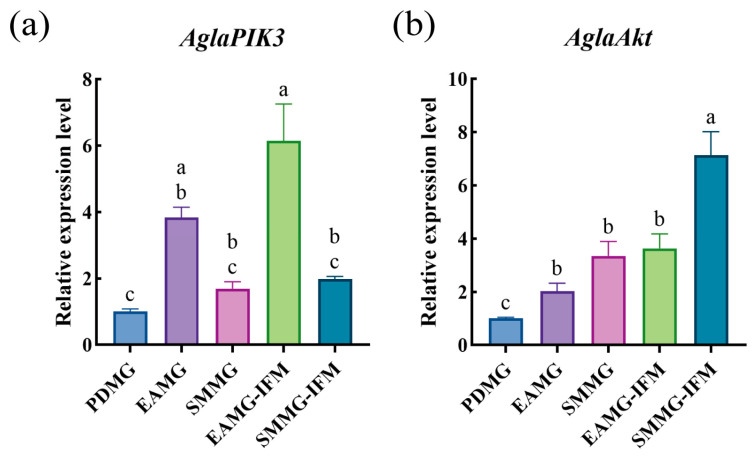
Expression profiles of *AglaPIK3* and *AglaAkt* in different treatment groups were analyzed based on qRT-PCR. (**a**) *AglaPIK3*; (**b**) *AglaAkt*. Data are expressed as mean  +  standard error (SEM) of three biological replicates. Different lowercase letters indicate significant differences among treatments (*p* < 0.05) as determined by one-way ANOVA with multiple comparisons performed using Tukey’s test.

Additionally, we measured the relative gene expression levels in the midguts of ALB adults that had fed on EA and SM for the same duration as the PDS-fed group. As shown in Figure 4, the expression of *AglaAkt* was significantly lower in beetles fed on PDS compared to those fed on the other two tree species.

### 3.4. Functional Analysis of AglaAkt Genes by RNAi Silencing

**Figure 5:**
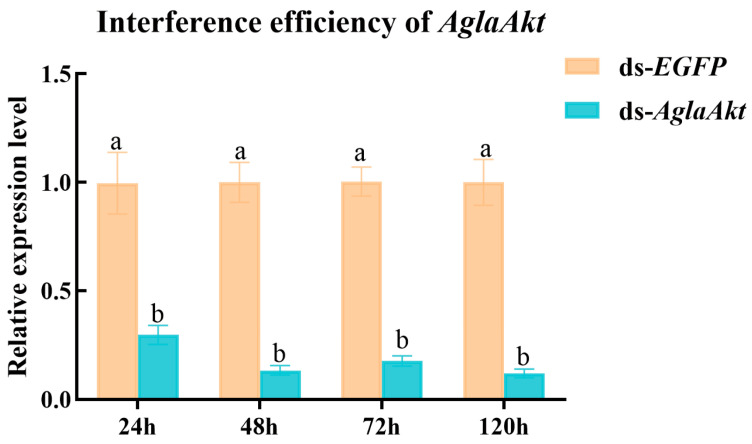
The relative expression levels of *AglaAkt* at 24 h, 48 h, 72 h, and 120 h after ds-*AglaAkt* injection were measured. ds-*EGFP* (enhanced green fluorescent protein) was used as a control. Data are expressed as mean  ±  SE of three biological replicates. Different lowercase letters indicate significant differences among treatments (*p* < 0.05), as determined by an independent Student’s *t*-test.

**Figure 6:**
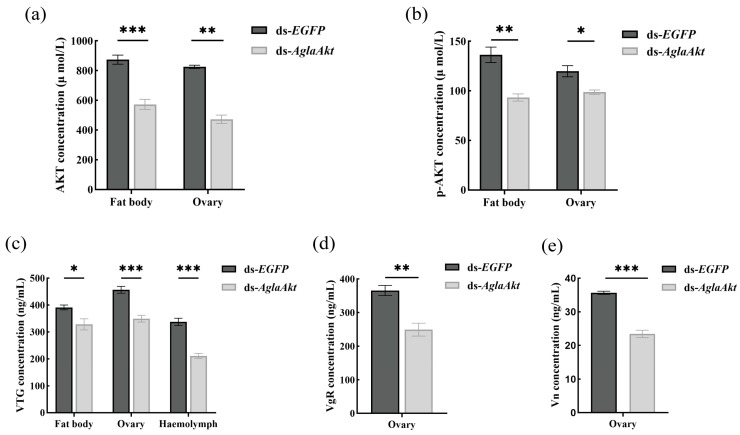
Changes in physiological parameters in *A. glabripennis* females 120 h after injection of ds-*AglaAkt*. (**a**) AKT protein/kinase (AKT); (**b**) phospho-AKT (p-AKT); (**c**) vitellogenin (VTG); (**d**) vitellogenin receptor (VgR); (**e**) vitellin (Vn). ds-*EGFP* was used as a control. Data are expressed as mean  ± SE of four biological replicates. The asterisk indicates a significant difference between treatment and control groups (* *p* < 0.05, ** *p* < 0.01, *** *p* < 0.001. Independent-sample *t*-test).

To further elucidate the functional role of the *AglaAkt* gene in response to ALBs exposed to PDS, RNA interference was employed to knockdown its expression in female adults. Quantitative analysis revealed a significant reduction in *Akt* gene transcript levels following ds-*AglaAkt* injection, with decreases observed at 24 h (to 29.56%, *p* < 0.05), 48 h (to 13.32%, *p* < 0.01), 72 h (to 17.60%, *p* < 0.01), and 120 h (to 11.83%, *p* < 0.001) compared to the control group (Figure 5). These results demonstrated effective and sustained gene silencing throughout the experimental period. We next measured the levels of AKT protein/kinase (AKT), phospho-AKT (p-AKT), vitellogenin (VTG), vitellogenin receptor (VgR), and vitellin (Vn) in ALBs following *AglaAkt* knockdown to explore its role in reproduction. At 120 h post ds-*AglaAkt* injection, significant reductions in AKT content were observed in the female fat body (34.67% decrease; t = 6.280, df = 6, *p* < 0.001) and ovaries (43.05% decrease; t = 9.731, df = 3.316, *p* < 0.01) compared to the control (Figure 6a). Similarly, p-AKT levels decreased by 31.39% (t = 5.550, df = 6, *p* < 0.01) in the fat body and 17.36% (t = 3.693, df = 6, *p* < 0.05) in the ovaries (Figure 6b). Vitellogenin content was also significantly reduced in the fat body (16.10% decrease; t = 2.793, df = 6, *p* < 0.05), ovaries (23.56% decrease; t = 6.033, df = 6, *p* < 0.001), and hemolymph (37.55% decrease; t = 8.186, df = 6, *p* < 0.001) relative to the ds-*EGFP*-injected controls (Figure 6c). Consistent with these findings, the levels of vitellogenin receptor and vitellin in the ovaries were reduced by 32.27% (t = 4.591, df = 6, *p* < 0.01; Figure 6d) and 34.50% (t = 8.655, df = 6, *p* < 0.001; Figure 6e), respectively.

## 4. Discussion

The Asian longhorn beetle is a destructive wood-boring pest that has attracted widespread attention and research. However, controlling it is challenging due to its unique growth and damage traits. Due to advancements in chemical ecology and molecular biology, increasing research suggests that pest control strategies using resistant plants or RNAi are feasible [50,51,52,53]. Previous studies have shown that female ALBs fed on PdS have shorter lifespans than females fed on EA or SM [37,39]. In this study, we used transcriptomic sequencing and qRT-PCR to analyze the midguts of female ALB fed on three different tree species, aiming to understand their molecular responses to various tree species and to identify how resistant poplar PdS affects ALB.

Transcriptomic results showed that comparing PdS-fed females to EA-fed females identified more DEGs than comparing PdS-fed females to SM-fed females (PDMG vs. EAMG and PDMG vs. SMMG, Figure 1c). This may also be due to the varying nutritional conditions that different plants provide to ALB [35,38]. Interestingly, the PIK3-Akt signaling pathway was enriched in the KEGG pathway analysis of DEGs in both comparison groups. In addition, the protein digestion and absorption pathway was significantly enriched in the KEGG enrichment analysis. Therefore, we selected six genes potentially related to nutrient absorption and reproduction—*AglaInSR*, *AglaPIK3*, *AglaAkt*, *AglaSLC15A1*, *AglaSLC36A-1*, and *AglaSLC36A-2*—for qRT-PCR validation. The results showed that the expression levels of the three solute carrier genes, *AglaSLC15A1*, *AglaSLC36A-1*, and *AglaSLC36A-2*, were higher in the midgut of ALBs feeding on PdS than in those feeding on the other two trees (Figure 3). However, given their indirect detoxification and metabolic functions, we consider including male adults in future studies to jointly analyze the role of this gene family in ALB responses to different plants. Further analysis indicated that the PIK3-Akt signaling pathway was suppressed in ALBs responding to PdS compared with ALBs responding to EA or SM. Specifically, the expression levels of *AglaAkt* in the midgut of ALBs fed on PdS were significantly lower than in those fed on SM and EA (Figure 4b). For the two upstream genes *AglaInSR* and *AglaPIK3* of *AglaAkt,* the differences in the midgut of ALBs feeding on different plants were not as significant as those of *AglaAkt* in this study.

As an essential downstream component of the insulin-like signaling pathway (ILP) and the PIK3-Akt signaling pathway, the *Akt* gene has been studied in various ways [23,54,55]. It has been studied across various insects, with results indicating that gene inhibition can shorten lifespan or regulate reproduction [16,17,56,57]. Furthermore, because conserved nutrient-sensitive pathways play an important role in transmitting nutrient signals and maintaining body homeostasis [58,59], a previous study has shown that expression of this gene in the digestive system can also affect insect reproductive function [56]. However, its function in the ALB and its potential as a form of pest control has not been explored. Because of the different expression patterns of *AglaAkt* across tree species, which might affect the lifespan and reproductive capacity of ALB, we then knocked down *AglaAkt* in ALBs by injecting dsRNA. Silencing the *AglaAkt* gene in adult females reduced AKT and p-AKT levels, resulting in significantly decreased VTG and VgR levels (Figure 6a–d), thereby reducing vitellin deposition in ALB ovaries. This is consistent with findings in *Cyrtorhinus lividipennis* Reuter (Hemiptera: Miridae) [18], *L. serricorne* [22,60], and *F. occidentalis* [23]. These results provide strong evidence that *AglaAkt* decreases the fertility of the ALB by affecting vitellogenin production and vitellin deposition.

Insect reproduction is a complex process requiring the participation of multiple factors, including juvenile hormones [7,16], 20E [61], insulin [62,63], and carbohydrates [64]. However, these physiological and biochemical substances can be altered with variations in food or nutritional conditions [57,65]. Based on this phenomenon, we could infer that longhorn beetles feeding on PdS may be in a nutritionally deficient stage compared to those feeding on SM or EA, thus affecting the mRNA level of the *AglaAkt* gene. This is similar to previous studies in *Drosophila melanogaster* Meigen (Diptera: Drosophilidae) [66] and *Liposcelis entomophila* (Enderlein) (Psocoptera: Liposcelididae) [16], where *Akt* downregulation leads to insufficient nutrition for oogenesis and embryonic development. Furthermore, a recent study showed that transgenic rice expressing a dsRNA fragment targeting *SfAkt* can silence the *SfAkt* gene in brown planthoppers, reduce vitellogenin expression, cause ovarian tubule atrophy, and inhibit egg development [20]. Therefore, we speculate that the *AglaAkt* gene may also be a key gene that contributes to ALB’s low reproductive capacity when feeding on resistant poplar PdS.

This study elucidated the molecular response patterns of female ALBs to different host plants by sequencing midgut transcriptomes of ALBs feeding on those hosts. Furthermore, RNAi was used to silence the *AglaAkt* gene to clarify its role in response to different hosts and in female reproduction. Therefore, on the one hand, the *AglaAkt* gene can regulate the reproductive function of ALB females by influencing AKT, p-AKT, VTG, VgR, and Vn levels. On the other hand, the downregulation of the *AglaAkt* gene in the midgut of ALBs feeding on resistant poplar PdS can indirectly explain the insect-resistant effect of PdS on ALBs. Although we have preliminarily investigated the gene function of *AglaAkt* in ALB, many challenges and limitations remain in studying the interactions between ALB and different plants, as well as the application of ds-*AglaAkt*. Therefore, future research should not only focus on its application evaluation after silencing via feeding and the optimization of dsRNA delivery methods, but also on investigating the response patterns of different ALB tissues across different plants (especially poplars with different resistance) to obtain a more comprehensive transcriptional profile for screening target genes and developing new control strategies. Collectively, these findings provide important insights into the role of the *AglaAkt* gene in mediating both reproductive development in ALB and its molecular interaction with the resistant poplar PdS.

## Figures and Tables

**Table 1:** Information of the female *Anoplophora glabripennis* midgut transcriptome.

Sample	Raw Reads	Clean Reads	GC (%)	Q20 (%)	Q30 (%)	Mapping to Reference Genome		
						Multiple Mapped	Uniquely Mapped	Total Mapped
PDMG1	44,683,084	44,383,020	45.11	98.76	96.16	1,983,121 (4.47%)	37,411,328 (84.29%)	39,394,449 (88.76%)
PDMG2	49,289,958	48,956,764	44.99	98.77	96.18	2,088,768 (4.27%)	40,934,594 (83.61%)	43,023,362 (87.88%)
PDMG3	40,644,308	40,351,662	44.83	98.73	96.07	1,965,988 (4.87%)	34,114,296 (84.54%)	36,080,284 (89.41%)
EAMG1	50,824,802	50,515,214	46.98	98.68	95.98	2,331,646 (4.62%)	35,935,563 (71.14%)	38,267,209 (75.75%)
EAMG2	46,919,610	46,608,834	46.8	98.65	95.92	2,308,850 (4.95%)	33,661,412 (72.22%)	35,970,262 (77.17%)
EAMG3	47,155,346	46,853,374	46.82	98.67	95.93	2,160,115 (4.61%)	31,832,037 (67.94%)	33,992,152 (72.55%)
SMMG1	42,843,542	42,535,010	45.58	98.67	95.96	2,576,238 (6.06%)	35,805,539 (84.18%)	38,381,777 (90.24%)
SMMG2	47,580,198	47,258,188	45.7	98.69	95.99	2,833,450 (6.0%)	40,003,547 (84.65%)	42,836,997 (90.64%)
SMMG3	46,181,186	45,836,158	45.47	98.7	96.04	2,514,061 (5.48%)	39,195,251 (85.51%)	41,709,312 (91.0%)

## Data Availability

The data supporting the findings of this study are available from the corresponding author upon reasonable request. The transcriptomic data can be retrieved from National Center for Biotechnology Information (NCBI) Sequence Read Archive (SRA) under the BioProject number PRJNA1391023 with the accession SRR36516247–SRR36516255.

## Supplementary Materials

The following supporting information can be downloaded at: https://www.mdpi.com/article/10.3390/insects17030250/s1, Figure S1: Quality control of transcriptome data for 9 *Anoplophora glabripennis* samples; Figure S2: Statistical analysis of GO classification of all DEGs across different treatment groups; Table S1: The primer sequence information for qRT-PCR and dsRNA synthesis; Table S2: TPM values of three comparison groups differentially expressed genes; Table S3: level-2 GO functional classifications of differentially expressed genes in each experimental group of the midgut of *A. glabripennis* adults; Table S4: KEGG significantly enriched pathways of differentially expressed genes in each experimental group of the midgut of *A. glabripennis* adults.
