# Supplementary figures and images for: Insect-Resistant Variety Populus deltoides ‘Shalinyang’ May Decrease Anoplophora glabripennis Females’ Fecundity by Suppressing the Serine/Threonine Kinase AglaAkt Gene
Source: Insects. 2026 Feb 27;17(3):250. doi: 10.3390/insects17030250 (PMC13027010; doi:10.3390/insects17030250)

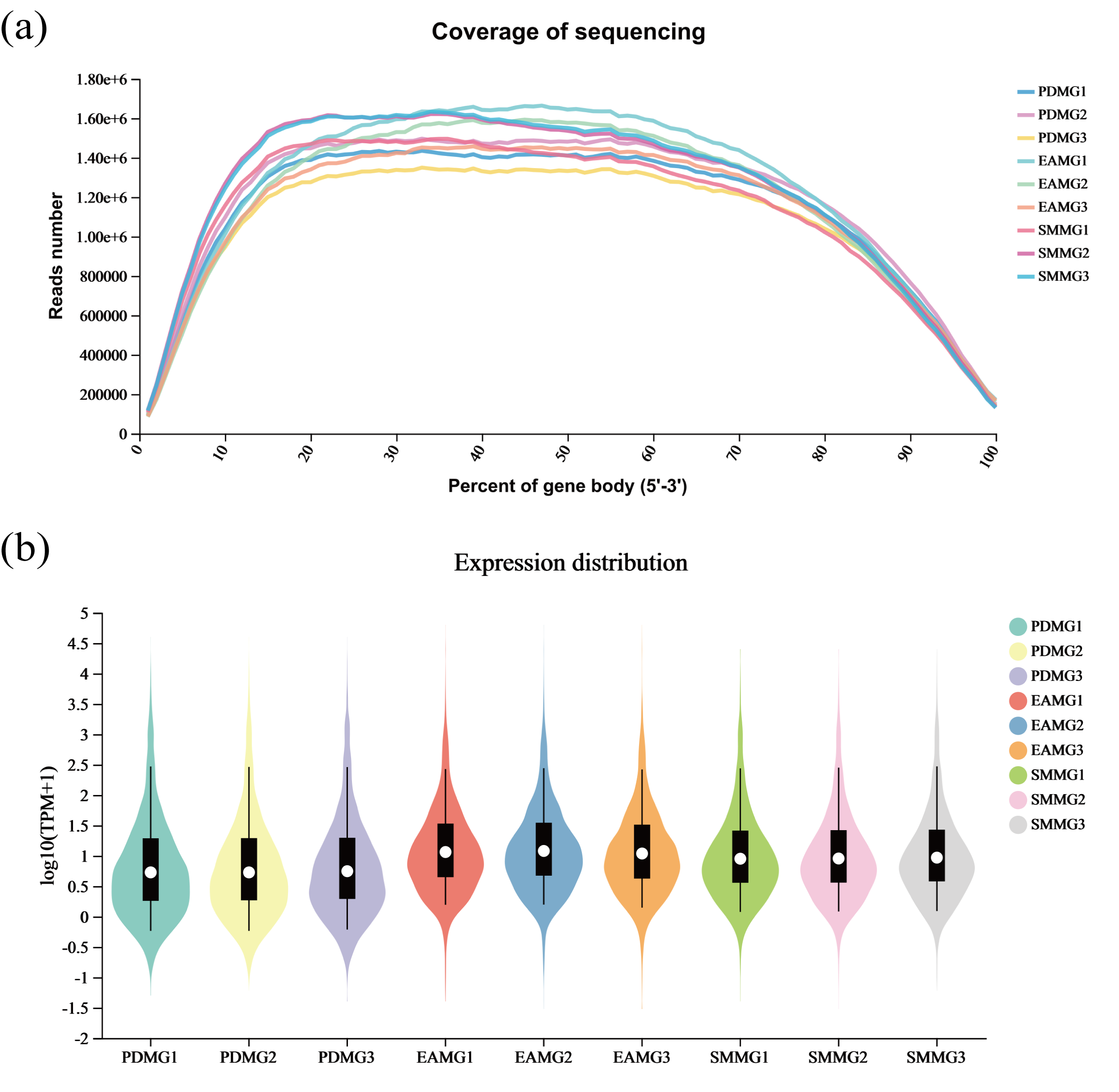

Supplement: Supplementary file 1 [file insects-17-00250-s001.zip › FigureS1.png]

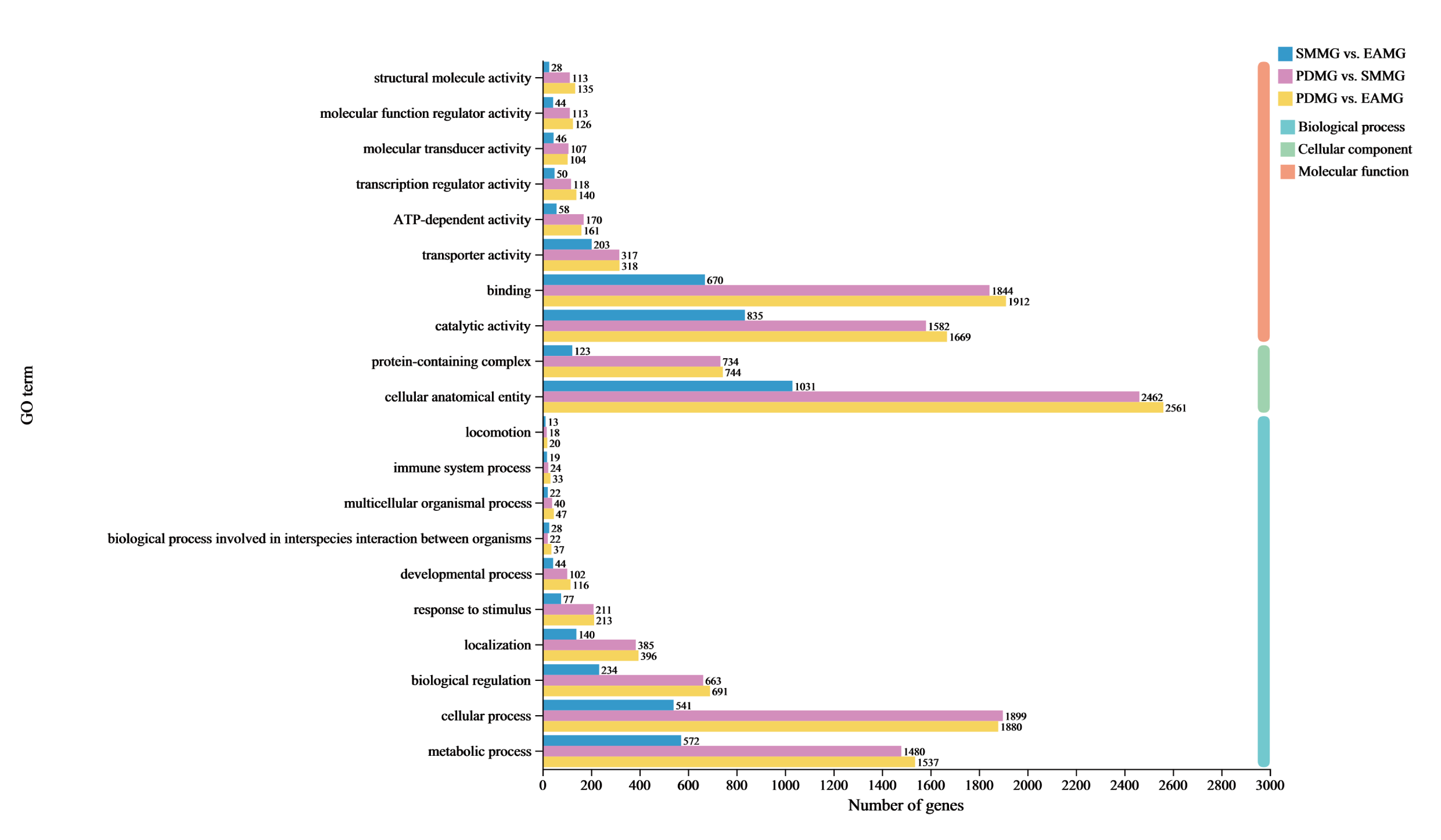

Supplement: Supplementary file 1 [file insects-17-00250-s001.zip › FigureS2.png]
